# Prevalence of child maltreatment in a nationwide sample of 18 to 31 year-olds in Germany

**DOI:** 10.1186/s13034-024-00795-z

**Published:** 2024-09-03

**Authors:** Christoph Kasinger, Robert Schlack, Elmar Brähler, Jörg M. Fegert, Vera Clemens

**Affiliations:** 1 Department for Psychosomatic Medicine and Psychotherapy, University Medical Center of Johannes Gutenberg University of Mainz, Untere Zahlbacher Str. 8, 55131 Mainz, Germany; 2 Department of Epidemiology and Health Monitoring, Robert Koch Institute, Berlin, Germany; 3 Department of Psychiatry and Psychotherapy, Leipzig University Hospital, Leipzig, Germany; 4 Department for Child and Adolescent Psychiatry/Psychotherapy, University of Ulm, Ulm, Germany; 5 German Center for Mental Health (DZPG), partner site Ulm, Ulm, Germany

**Keywords:** Child maltreatment (CM), Prevalence, CTQ, Childhood, Abuse, Neglect, Household dysfunctions, SDG 16.2.3

## Abstract

**Background:**

Child maltreatment (CM) can have devastating and potentially lifelong effects for those affected and is a major contributor to mental health problems. To tackle public health problems it is crucial to have reliable data on CM. The aim of this study is to assess the prevalence and predictors of CM in a nationwide sample of the German population of young adults.

**Methods:**

The study population (young adults aged 18 to 31 years) stems from the KiGGS Cohort study, the longitudinal branch of the German Health Interview and Examination Survey for children and adolescents. This sample meets the criteria of the United Nations Sustainable Development Goals (SDG) indicator 16.2.3. The data was collected between 2014 and 2017. CM were assessed with the Childhood Trauma Questionnaire (CTQ) in. In addition, socio-demographic variables and other known risk factors for CM were assessed. A total of 6433 (47.8% female) participants were included in the analyses. Binary logistic regression analyses were used to investigate predictors of maltreatment subtypes. Ordinal regression was used to examine their association with experience of multiple forms of CM.

**Results:**

Overall, 18.4% (f: 20.9%, m: 16.1%) of the participants reported having experienced at least one type of CM; 6.7% (f: 8.8%, m: 4.8%) reported experiences of emotional abuse, 3.7% (f: 3.9%, m: 3.5%) physical abuse, 3.5% (f: 5.3%, m: 1.7%) sexual abuse, 9.0% (f: 9.9%, m: 8.2%) emotional neglect and 8.6% (f: 8.5%, m: 8.7%) physical neglect. Gender, subjective social status, education and household dysfunction (e.g. living with an individual who is using substances) emerged as significant predictors for different types of CM. Additionally, all these factors were significant risk factors for experiencing cumulative CM.

**Conclusions:**

CM is common in the German population, with almost one in five people experiencing at least one type of CM. The results reveal important risk factors for the occurrence of CM. In particular, people with lower social status and those who grew up in dysfunctional households are at higher risk of CM. Greater support for this vulnerable population may reduce the prevalence of CM.

## Introduction

Child maltreatment (CM) has been linked to a significant burden of physical and mental health issues across the lifespan [1, 2], with studies indicating a potential reduction in life expectancy of up to 20 years [3]. Besides these severe consequences for affected individuals, the economic costs are considerable. In Germany, for instance, the estimated annual expenses resulting from CM are between 11 and 30 billion Euros [4]. Unfortunately, CM is highly prevalent: For example, in Germany, approximately 13% of participants in a nationwide study have reported an incident of child sexual abuse [5]. Consequently, CM represents a significant public health issue.

The rights-based Sustainable Development Goals (SDGs), which were ratified by all UN member states in 2015, include the end of abuse, exploitation, trafficking and all forms of violence against and torture of children as a key sustainability goal (SDG 16.2). The United Nations formulated the survey of the incidence of CM before the age of 18 as indicator 16.2.1 and the proportion of young people aged 18–29 years who experienced sexual violence by age 18 as indicator 16.2.3. As it is suggested that about 90% of cases of CM go undetected [6], population-based data beyond official reports is of particular relevance.

To date, population-based research on the prevalence of CM in Germany has focused on samples comprising individuals across the entire adult range, extending up to 90 years of age [7–9]. Two studies conducted in 2010 and 2016 utilising the Childhood Trauma Questionnaire (CTQ), yielded prevalence rates for moderate to severe forms of CM between 4.6% and 6.5% for emotional abuse, 5.6% and 6.7% for severe physical abuse and 6.2% and 7.6% for sexual abuse. Prevalence rates of moderate to severe neglect were between 13.3% and 13.9% for emotional neglect and between 22.5% and 28.8% for physical neglect [7, 9]. Subsequent studies utilising comparable samples have demonstrated variablitiy in prevalence rates in dependence of the employed measure. Higher rates have been observed for abusive forms of CM, while lower rates have been documented for neglect [8, 10]. These findings are more aligned with international data [11].

As all these studies focused on samples with the majority of participants older than 30 years, they do not meet the criteria of SDG indicator 16.2.3. The assessment of CM in younger age groups allows for the estimation of the prevalence of CM over the last two decades in Germany. Witt et al. conducted an analysis of subsamples from a population-based sample, focusing on the age group 18–29 years [5]. However, the size of the subsample was relatively small, comprising fewer than 400 individuals. Furthermore, only sexual abuse was assessed. Using the same data as the present study, Cohrdes and Mauz [12] examined the mediating role of self-efficacy and emotional stability in the association between childhood maltreatment and health-related quality of life. They also provide an overview of the frequency of CM. However, the population studied is different and much smaller than the present study population. In addition, the classification of CM does not follow the common international classification and therefore lacks comparability.

Consequently, the present study aimed to assess the prevalence of CM in a nationwide sample of the German population aged 18–31 years, thereby providing initial German data for monitoring CM in accordance with the UN SDGs.

## Methods

### Sample

The data for this study is based on a representative survey conducted by the Robert Koch Institute as part of the “Health of Children and Adolescents in Germany” (KiGGS) study. Participants were recruited using a two-stage selection process, involving random sampling from local registration offices. The survey includes a baseline survey, which was conducted between 2003 and 2006, and two follow-up surveys, enabling cross-sectional and longitudinal analyses. The data employed in this article is derived from the longitudinal second follow-up (KiGGS Cohort), which was obtained through written postal questionnaires between 2014 and 2017 [13]. Of the original 17,641 respondents from the baseline survey, a total of 10,853 individuals could be reached again, resulting in a response rate of 61.5%. Only adult individuals (aged 18 years or older) were included in the present study as the CTQ was administered to adult individuals only. In order to make more representative statements, a weighting factor provided by the authors of the KIGGS study was applied to correct for sample deviations present in the sample. The weighting factor adjusts the sample regard to age, gender, federal state, nationality and parental educational attainment to the official population statistics as at 31 December 2015 [14]. Furthermore, the weighting factor accounts for panel attrition effects, given that the KiGGS survey is under-represented in terms of participants from lower social backgrounds, older age groups and those with an immigrant background [15]. This resulted in a final adjusted sample size of *N* = 6,433 (Mean age = 23.74, Age range = 18 to 31 years, Birth years: 1985–1999, Female = 47.80%). Further sociodemographic characteristics are presented in Table 1.

**Table 1:** Sociodemographic characteristics

	Total (*N* = 6,433)	Female (*N* = 3,076)	Male (*N* = 3,356)
Age; mean (SD) Age; range	23.74 (3.37) 18–31	23.75 (3.35) 18–31	23.74 (3.39) 18–31
Highest level of education			
No school diploma	209 (3.3%)	89 (2.9%)	120 (3.7%)
Lower secondary school certificate	3,020 (48.2%)	1,345 (44.4%)	1,675 (51.8%)
A-Level Certificate	3,020 (48.2%)	1,600 (52.7%)	1,420 (43.9%)
Subjective social status			
Low	3,126 (51.3%)	1,546 (52.4%)	1,581 (50.3%)
High	2,966 (48.7%)	1,403 (47.6%)	1,563 (49.7%)
Living with substance abuser	877 (14.0%)	523 (17.3%)	354 (10.9%)
People in household depressed/suicide attempt	873 (14.0%)	475 (15.8%)	398 (12.4%)
Family member was/is in jail	303 (4.9%)	164 (5.4%)	140 (4.4%)
Experience of war/terrorism/conflicts	81 (1.5%)	30 (1.1%)	51 (1.8%)

*Note.* Missing values for Education = 2.6%, subjective social status = 5.3%, living with substance abuser = 2.6%, people in household depressed/suicide attempt = 3.1%, family member in jail = 3.6%, experience of war/terrorism/conflicts = 14.2%

## Measures

CM was assessed using the Childhood Trauma Questionnaire (CTQ) [16–18]. The CTQ comprises five subscales, namely sexual, emotional and physical abuse, as well as emotional and physical neglect. Each subscale consists of five items, with a five point-Likert response format. The subscale scores are calculated using the sum score of the five items. The German version of the CTQ has been demonstrated to possess valid and reliable psychometric properties by Klinitzke and colleagues [16]. The internal consistencies for all subscales ranged between 0.62 and 0.96 and the intra-class coefficient for an interval of six weeks was 0.77 for the overall scale and between 0.58 and 0.81 for subscales. Cronbachs α ranged from good to excellent (0.76 and 0.94) in our sample for all CTQ subscales. Only the subscale physical neglect showed an insufficient internal consistency of α = 0.40.

The severity scores for each subscale were calculated based on norm data provided by Haeuser et al. [9]. The severity scores rang from “none-minimal”, “minimal-moderate”, “moderate-severe”, to “severe-extreme”. Dichotomous scores (e.g. experience of emotional neglect: yes/no) were based on scores reaching at least the moderate-severe level ( > = 13 for emotional abuse, >= 10 for physical abuse, >=8 for sexual abuse, >=15 for emotional neglect and > = 10 for physical neglect). Cumulative CM was calculated in accordance with the number of CTQ subscales that were reported as “moderate-severe” or “severe-extreme”.

Gender, living with an individual engaged in substance misuse, family member in household that is depressed/attempted suicide, family member that was/is in jail and experience of war/terrorism/conflicts were considered as predictors in our analysis and were obtained using a dichotomous response format (e.g. Did you live with an individual in your household who was engaged in substance misuse? Yes/no). Education was operationalized by asking for the highest school certificate. The information was then dichotomised, whereby holding an A-level certificate (equivalent to a high school diploma) was coded as 1 and holding no A-level certificate was coded as 0.

Subjective social status was measured with the MacArthur Scale of Subjective Social Status [19]. This is a single item question, in which respondents view a drawing of a ladder with 10 rungs. The introduction reads as follows: “At the top of the ladder are the people who are the best off, those who have the most money, most education, and best jobs. At the bottom are the people who are the worst off, those who have the least money, least education, worst jobs, or no job. Please place an ‘X’ on the rung that best represents where you think you stand on the ladder.”

The response scale ranges from 1 to 10. A binary transformation was performed, whereby values from 1 to 5 were classified as low subjective social status, while values from 6 to 10 were classified as high subjective social status.

### Statistical analyses

**Fig. 1:**
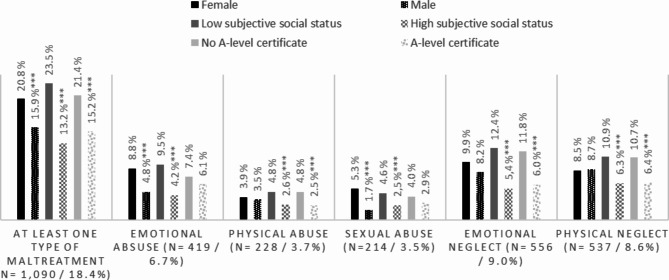
Prevalence of CM by socio-demographic variables.  * = p < .05; ** = p  < .01; ***= p  < .001. Significance testing using Chi-square tests has been performed separately for gender, subjective social status and education

All analyses were conducted with SPSS version 26. The Prevalence rates are presented in accordance with the four severity categories stratified by gender. Additionally, the prevalence rates for participants who experienced at least moderate to severe maltreatment are reported. The prevalence rates for the experience of at least moderate to severe maltreatment stratified by different socioeconomic characteristics are presented in Fig. 1. For these comparison testings Chi-Square-tests were conducted. To account for α-inflation, a Bonferroni correction was conducted for the three tests within each subtype (18 tests). In Table 3 the co-occurence of subtypes of childhood maltreatment are presented.

Odds ratios with corresponding 95% confidence intervals for the various predictors were calculated using five binary logistic regression analyses, with all five dichotomised subtypes of childhood maltreatment as dependent variables. The role of gender, subjective social status, education and adverse living conditions (e.g. living with an individual engaged in substance misuse) were investigated as predictors. Furthermore, an ordinal regression analysis was conducted to ascertain the significance of the above mentioned predictors with respect to cumulative maltreatment experiences. The count of instances of each subtype of childhood maltreatment was used as dependent variable. Unstandardized beta coefficient and the corresponding 95% confidence interval for the ordinal regression analysis are reported herewith. We followed a complete case approach which means only cases with no missing values for any of the variables were included in the analysis.

## Results

Of our sample 18.4% (1,121 individuals) reported to have experienced at least one form of CM in a at least moderate to severe form. In terms of specific CM types among all participants, 6.7% reported experiencing at least moderate emotional abuse, 3.7% reported at least a moderate instance of physical abuse, 3.5% reported incidents of sexual abuse, 9.0% acknowledged emotional neglect, and 8.6% experienced at least a moderate form of physical neglect. Detailed results are presented in Table 2.

**Table 2:** Prevalence rates of CM

	*N*	None to minimal *N* (%)	Low–moderate *N* (%)	Moderate to severe *N* (%)	Severe to extreme *N* (%)	Experience of at least moderate to severe CM *N* (%)
Emotional Abuse						
Total	6,225	4,943 (79.4)	863 (13.9)	214 (3.4)	205 (3.3)	419 (6.7)
Female	3,022	2,290 (75.8)	466 (15.4)	125 (4.1)	141 (4.7)	266 (8.8)
Male	3,203	2,653 (82.8)	397 (12.4)	89 (2.8)	64 (2.0)	153 (4.8)
Physical Abuse						
Total	6,223	5,745 (92.3)	248 (4.0)	136 (2.2)	92 (1.5)	228 (3.7)
Female	3,014	2,777 (92.1)	118 (3.9)	67 (2.2)	52 (1.7)	119 (3.9)
Male	3,209	2,968 (92.5)	130 (4.1)	69 (2.2)	42 (1.3)	111 (3.5)
Sexual Abuse						
Total	6,194	5,829 (94.1)	151 (2.4)	140 (2.3)	74 (1.2)	214 (3.5)
Female	3,015	2,732 (90.6)	122 (4.0)	106 (3.5)	55 (1.8)	161 (5.3)
Male	3,179	3,097 (97.4)	29 (0.9)	34 (1.1)	19 (0.6)	53 (1.7)
Emotional Neglect						
Total	6,205	4,461 (71.9)	1188 (19.1)	295 (4.8)	261 (4.2)	556 (9.0)
Female	2,996	2,154 (71.9)	547 (18.3)	140 (4.7)	155 (5.2)	295 (9.9)
Male	3,209	2,307 (71.9)	641 (20.0)	155 (4.8)	106 (3.3)	261 (8.2)
Physical Neglect						
Total	6,222	4,781 (76.8)	907 (14.6)	348 (5.6)	189 (3.0)	537 (8.6)
Female	3,017	2,358 (78.2)	404 (13.4)	162 (5.4)	93 (3.1)	255 (8.5)
Male	3,205	2,423 (75.6)	503 (15.7)	186 (5.8)	93 (2.9)	279 (8.7)

Number of experienced forms of CM	Total (*N* = 6,096)	Female (*N* = 3,138)	Male (*N* = 2,958)
0	4,975 (81.6%)	2,341 (79.1%)	2,634 (83.9%)
1	649 (10.6%)	340 (11.5%)	309 (9.8%)
2	272 (4.5%)	146 (4.9%)	126 (4.0%)
3	125 (2.0%)	77 (2.6%)	48 (1.5%)
>= 4	75 (1.2%)	54 (1.8%)	21 (0.6%)

### Predictors of childhood maltreatment

Prevalence rates of CM by socio-demographic variables (see Fig. 1).

Female participants (χ² = 23.352, df = 1, *p* < .001), participants with a low subjective social status (χ² = 103.176, df = 1, *p* < .001) and participants who do not hold an A-level certificate (χ² = 38.246, df = 1, *p* < .001) reported higher rates of at least one type of maltreatment. Females were affected by *emotional abuse* more often than males (χ² = 32.598, df = 1, *p* < .001). Additionally, those with low subjective social status reported a higher prevalence of emotional abuse compared to those with high subjective social status (χ² = 65.182, df = 1, *p* < .001). There was no gender difference pertaining to the prevalence of *physical abuse*, however, participants with low subjective social status displayed a higher prevalence of physical abuse compared to those with high subjective social status (χ² = 21.369, df = 1, *p* < .001). Similarly, participants without an A-level certificate had a higher prevalence of physical abuse compared to those with an A-level certificate (χ² = 22.997, df = 1, *p* < .001). Females reported a higher prevalence of *sexual abuse* compared to males (χ² = 62.538, df = 1, *p* < .001). Additionally, those with low subjective social status were also more affected by sexual abuse than those with high subjective social status (χ² = 19.540, df = 1, *p* < .001). Participants with low subjective social status also had a notably higher prevalence of *emotional neglect* than those with high subjective social status (χ² = 92.417, df = 1, *p* < .001). Similarly, participants without an A-level certificate had a higher prevalence of emotional neglect compared to those with an A-level certificate (χ² = 62.373, df = 1, *p* < .001). Similar trends emerged for *physical neglect*: participants with low subjective social status reported physical neglect more often than those with high subjective social status (χ² = 40.043, df = 1, *p* < .001). Likewise, participants without an A-level certificate had a higher prevalence of physical neglect compared to those with an A-level certificate (χ² = 35.202, df = 1, *p* < .001).

### Co-occurence of types of CM

Overall, 57.71% (*n* = 647 out of 1,121.) of all individuals with an experience of child maltreatment experienced only one type of maltreatment. With regard to the single types of abuse 25.30% (*N* = 106 out of 419) of those reporting emotional abuse, 18.86% (*N* = 43 out of *N* = 228) of those reporting physical abuse, 45.33% (*N* = 97 out of *N* = 214) of those reporting sexual abuse, 33.27% (*N* = 185 out of *N* = 556) of those reporting emotional neglect, and 40.22% (*n* = 216 out of *N* = 537) of those reporting physical neglect indicated no other type of maltreatment.

The most prevalent combinations of maltreatment types were emotional abuse and emotional neglect (7.67%), emotional and physical neglect (7.14%), emotional abuse, emotional and physical neglect (3.93%), and combined emotional abuse, physical abuse, emotional neglect and physical neglect (3.12%). Having experienced all five types of maltreatment was reported from 1.16% of the participants with at least one type of experienced maltreatment. For detailed information see Table 3.

**Table 3:** Co-occurence of types of CM

Type/combination of CM	*N*	Percentages in relation to individuals with at least one type of CM (*N* = 1,121)
Emotional abuse (EA) only	106	9.46
Physical abuse (PA) only	43	3.84
Sexual abuse (SA) only	97	8.65
Emotional neglect (EN) only	185	16.50
Physical neglect (PN) only	216	19.27
EA + PA	21	1.87
EA + SA	6	0.54
EA + EN	86	7.67
EA + PN	15	1.34
PA + SA	6	0.54
PA + EN	15	1.34
PA + PN	18	1.61
SA + EN	8	0.71
SA + PN	19	1.70
EN + PN	80	7.14
EA + PA + SA	9	0.80
EA + PA + EN	23	2.05
EA + PA + PN	9	0.80
EA + SA + EN	4	0.36
EA + SA + PN	12	1.07
EA + EN + PN	44	3.93
PA + SA + EN	0	–
PA + SA + PN	2	0.18
PA + EN + PN	14	1.25
SA + EN + PN	8	0.71
EA + PA + SA + EN	3	0.27
EA + PA + SA + PN	7	0.62
EA + PA + EN + PN	35	3.12
EA + SA + EN + PN	17	1.52
PA + SA + EN + PN	0	–
EA + PA + SA + EN + PN	13	1.16

*Note*. EA = emotional abuse, PA = physical abuse, SA = sexual abuse, EN = emotional neglect, PN = physical neglect

### Predictors of CM

The Results for the multivariate analyses are presented in Table 4. Gender turned out to be a significant predictor of *emotional abuse*. Females had 1.88 times higher odds(95% CI = 1.49–2.37) of reporting emotional abuse compared to males. Participants with lower subjective social status, those living with a substance abuser, those with family members who had been incarcerated, and those who experienced war or terrorism were also at significantly higher risk of experiencing emotional abuse. The logistic regression model for *physical abuse* showed, that participants who hold an A-level certificate had significantly lower odds of experiencing physical abuse, while those who were living with substance abuser or people in the household who were depressed or attempted suicide and those with family members who had been incarcerated had significantly higher odds of reporting physical abuse. Interestingly, subjective social status was no significant predictor for sexual abuse anymore after controlling for other relevant variables. Gender emerged as a significant predictor of *sexual abuse* in the logistic regression model. Females had 4.48 times higher odds (95% CI = 3.07–6.54) of experiencing sexual abuse compared to males. Low subjective social status, living with substance abuser or with people who were depressed/attempted suicide and experiencing war/terrorism/conflicts were also significantly associated with increased odds of sexual abuse. Participants with lower subjective social status, those who did not hold an A-level certificate, those living with substance abuser or with people who were depressed or attempted suicide, and those with family members who had been incarcerated were at significantly higher risk of *emotional neglect.* In the logistic regression model of *physical neglect*, participants with a low subjective social status and those who had not graduated with an A-level certificate were at significantly higher risk of physical neglect. Conversely, living with substance abuser or living with someone who was depressed or attempted suicide and having family members who had been incarcerated were also associated with increased odds of physical neglect.

**Table 4:** Results of the adjusted binary logistic regression

		Odds ratio (OR)	95% Confidence interval (CI)	*p*
Emotional Abuse (*N* = 5,251)	Gender (ref = male)	1.88	1.49–2.37	< 0.001
	Subjective social status (ref = high status)	2.21	1.72–2.84	< 0.001
	A-level certificate (ref = yes)	0.90	0.71–1.13	0.358
	Living with substance abuser	2.18	1.70–2.81	< 0.001
	People in household depressed/suicide attempt	3.14	2.47–4.00	< 0.001
	Family member was/is in jail	1.74	1.22–2.48	0.002
	Experience of war/terrorism/conflicts	3.22	1.71–6.06	< 0.001
Physical Abuse (*N* = 5,262)	Gender (ref = male)	1.04	0.78–1.40	0.787
	Subjective social status (ref = high status)	1.25	0.91–1.72	0.161
	A-level certificate (ref = yes)	1.67	1.22–2.29	0.001
	Living with substance abuser	4.07	2.98–5.56	< 0.001
	People in household depressed/suicide attempt	2.22	1.61–3.05	< 0.001
	Family member was/is in jail	3.09	2.10–4.53	< 0.001
	Experience of war/terrorism/conflicts	1.47	0.57–3.81	0.426
Sexual Abuse (*N* = 5,229)	Gender (ref = male)	4.48	3.07–6.54	< 0.001
	Subjective social status (ref = high status)	1.80	1.30–2.51	0.001
	A-level certificate (ref = yes)	1.08	0.79– 1.49	0.616
	Living with substance abuser	2.10	1.50–2.94	< 0.001
	People in household depressed/suicide attempt	2.03	1.45–2.85	< 0.001
	Family member was/is in jail	1.72	1.07–2.85	0.240
	Experience of war/terrorism/conflicts	3.43	1.49–7.86	0.004
Emotional Neglect (*N* = 5,243)	Gender (ref = male)	1.08	0.89–1.32	0.420
	Subjective social status (ref = high status)	2.03	1.63–2.52	< 0.001
	A-level certificate (ref = yes)	1.71	1.39–2.12	< 0.001
	Living with substance abuser	2.14	1.71–2.68	< 0.001
	People in household depressed/suicide attempt	2.27	1.81–2.84	< 0.001
	Family member was/is in jail	1.89	1.37–2.61	< 0.001
	Experience of war/terrorism/conflicts	0.58	0.23–1.48	0.252
Physical Neglect (*N* = 5,256)	Gender (ref = male)	0.85	0.69–1.03	0.102
	Subjective social status (ref = high status)	1.45	1.17–1.79	0.001
	A-level certificate (ref = yes)	1.45	1.18–1.79	0.001
	Living with substance abuser	3.49	2.80–4.36	< 0.001
	People in household depressed/suicide attempt	1.64	1.30–2.08	< 0.001
	Family member was/is in jail	2.77	2.04–3.76	< 0.001
	Experience of war/terrorism/conflicts	0.84	0.38–1.90	0.679

### Predictors for cumulative CM

All variables in the ordinal regression model are significant predictors for cumulative CM. Being female, reporting a low subjective social status, having no A-level certificate, living in a household with an individual who is misusing substances, suffer from depression or attempted suicide, as well as having a family member, who was in jail or having experienced war, terrorism or conflicts are associated with a higher risk of experiencing multiple adverse childhood events. The results are presented in Table 5.

**Table 5:** Results of adjusted binary logistic regression analysis for cumulative forms of CM(*N =* 5,151)

	Unstandardized Beta Coefficient	95% Confidence intervall (CI)	*p*
Gender (ref = male)	0.29	0.14–0.44	< 0.001
Subjective social status (ref = high status)	0.58	0.43–0.74	< 0.001
A-level certificate (ref = yes)	0.26	0.11–0.42	0.001
Living with substance abuser	1.10	0.93–1.27	< 0.001
People in household depressed/suicide attempt	0.95	0.78–1.13	< 0.001
Family member was/is in jail	0.83	0.57–1.08	< 0.001
Experience of war/terrorism/conflicts	0.60	0.10–1.11	0.020

## Discussion

This is the first study in Germany to assess the prevalence of CM in a large-scale nationwide sample of young people between the ages of 18 and 31 years. Hence, our analysis provides first data for a systematic and regular recording of the frequency of violence against children and young people Thus, the results of this analysis may be used for establishing a preliminary foundation for for a respective monitoring in Germany as recommended in the SDG indicator 16.2 by the UN. Coninous data are crucial for the development of prevention and intervention strategies, serve to identify vulnerable groups of children and young people and may ensure the effectiveness of intervention measures.

The results indicate a high prevalence of CM among young people in Germany, with 18.4% of participants reporting at least one form of CM. Emotional neglect was the most frequently reported type, experienced by 9% of participants.

In our sample, the prevalence of CM, particularly in terms of physical and emotional neglect, was lower than that in previously assessed representative samples of the German population [7]. This may be attributed, at least in part, to the younger age profile of our sample given that rates of neglect were shown to be higher in the population above the age of 30 years [7]. The lower rates among younger age groups may be attributed to cohort effects, given the evident shift in parenting norms in Germany. For example, there has been a notable decline in the affirmation of corporal punishment in Germany. A survey conducted in 2007, revealed that 68% of parents had already resorted to corporal punishment [20]. In contrast, a survey conducted in 2016 indicated that only 44.5% of respondents affirmed the use of corporal punishment, with younger age groups exhibiting lower rates of affirmation [21]. This may be partly attributed to efforts of institutions such as the codification of the SDGs. Furthermore, since 2000, the use of violence against children has been prohibited by law [22].

In comparison toto the aforementioned data from representative samples of the German population between the age of 14 and 25 assessed in 2010 and 2016, the rates found in the present study are comparable with respect to physical and emotional abuse, but lower for sexual abuse as well as emotional and physical neglect [23]. However, the demonstrated lower rate for physical neglect is comparable to international data regarding the prevalence of physical neglect in Europe [11]. In the study conducted by Witt et al., only a subsample of the respective age group was assessed, resulting in a relatively low number of individuals and a consequent limitation in external validity. The present study thus adds to the existing literature by reporting the first results from a large population-based nationwide sample.

The results indicated that women were at a significantly higher risk for emotional and sexual abuse. This finding is consistent with existing literature indicating that the experience of CM is gender-dependent, particularly in the context of sexual abuse, where affected girls are more prevalent [8, 24]. A lower subjective social status was found to be associated with an elevated risk for all forms of CM, with the exception of physical abuse. The risk of CM was significantly increased in cases where the subjects were living with substance abuser, with persons in the household who are depressed or committed a suicide attempt or are/were incarcerated. Furthermore, the risk was elevated in instances where the subjects had experienced war, terrorism, or conflicts. These features represent established risk factors for CM. Felitti et al. demonstrated increased ratios of CM in case of a substance abuse and a mental illness of a household member as well as in households with an incarcerated member [25]. These risk factors, along with low socioeconomic status, have been repeatedly validated in international samples [26, 27] and national studies [28, 29] as major risk factors for CM. The relationship between these risk factors and CM is complex and interwoven. Socioeconomic status, isolation and stigma are risk factors for, and also a consequence of CM. They are also associated with household dysfunctions, as well as with parental mental illness and drug abuse [30–37]. Parenting skills and parent-child interactions may be impaired in parents with mental illness [38] and parents with substance misuse [39].This may be linked to the increased risk for CM. Despite the established association between these risk factors and CM, our findings reiterate the heightened risk for children in families affected by mental illness and/or substance misuse. The necessity for targeted support for families with parental mental illness and/or substance misuse is thus made apparent.

The major advantage of this study is the extensive sample size of our population-based cohort sample of young people in the age range of young adulthood. This allows for an estimation of the prevalence of CM over the past two decades in Germany, and subsequently, for the monitoring of CM in accordance with the recommendations outlined by the UN in the SDGs.

A primary limitation of this study is the retrospective assessment of CM., which may result in an underestimation of the prevalence of CM due to recall bias, shame and misunderstandings [40, 41]. A critical debate has been held on the validity of retrospectively assessed CM. It has been demonstrated that individuals who report CM retrospectively constitute a distinct group from those who report CM prospectively [42], Nevertheless, subjective reports of childhood maltreatment have been shown to be associated with adverse consequences for later health, making themhighly relevant for adulthood [43]. Consequently, although our study may identify different individuals than those who report CM prospectively, our data is of high relevance given that prospectively measured data of CM do not yet exist in population-based surveys in Germany. To gain insight into the prevalence of CM during a highly sensitive period, longitudinal assessments of CM in cohorts starting at birth are required. It has been demonstrated that a decline in recall accuracy may occur at the time of CTQ assessment, suggesting that an earlier assessment might result in less memory bias [44]. This may explain why the results in our relatively young sample are considered as more valid compared to older samples. To the best of our knowledge, no data on CM in such a large-scale representative sample of young people in Germany is currently available. Therefore, our findings are of high relevance. The scope of our analyses was to assess the prevalence rates of CM. The possible consequences of CM were not addressed in the present study. Despite the inclusion of well-established risk factors inour analyses, the etiology of CM was not considered in this study and should be addressed in future population-based surveys.

In conclusion, our data provide the first evidence from a large-scale nationwide sample for the prevalence of CM in young people between the ages of 18 and 31 years in Germany as suggested by the SDGs. The findings confirm results of earlier population-based studies, which demonstrated a high prevalence of CM in the German population. This evidence underscores the necessity for action to improve the protection of children and adolescents in Germany. Our findings reiterate the need of targeted support for families affected by parental mental illness and/or substance misuse. It is recommended that the burden and consequences of CM be addressed in the treatment of adult patients with mental health problems.

## Data Availability

Data and materials are available upon reasonable request from the corresponding author.
